# Ruminant meat flavor influenced by different factors with special reference to fatty acids

**DOI:** 10.1186/s12944-018-0860-z

**Published:** 2018-09-24

**Authors:** Muhammad Sajid Arshad, Muhammad Sohaib, Rabia Shabir Ahmad, Muhamad Tahir Nadeem, Ali Imran, Muhammad Umair Arshad, Joong-Ho Kwon, Zaid Amjad

**Affiliations:** 1 Institute of Home and Food Sciences, Government College University, Faisalabad, Pakistan; 2 Department of Food Science and Human Nutrition, University of Veterinary and Animal Sciences, Lahore, Pakistan; 3 School of Food Science and Biotechnology, Kyungpook National University, Daegu, South Korea

**Keywords:** Meat flavor, Pre- and postharvest factors, Fatty acids, Cooking methods

## Abstract

Ruminant meat flavor is an important quality and sensory parameter which relays mainly on the organoleptic characteristics of meat. Meat flavor is vital factor for the palatability and acceptability of meat by the consumers. There are various intrinsic and extrinsic factors that influence eating quality of meat. Among these factors, flavor is the major contributor. Fat and low-molecular-weight water-soluble compounds are the most important precursor components in meat, responsible for the meat flavor. The present review focus on the different pre and post-harvest factors that influences the ruminant meat flavor. Raw meat has little flavor but cooking adds value in flavor due to different temperature and cooking methods. The volatile flavoring compounds which are responsible for cooked meat flavor are produced thermally by the Maillard’s reaction itself or interaction with lipid oxidation products and vitamin degradation. In nutshell, this review provides perception into previous literature on flavor that affected by various factors particularly the fatty acids and cooking methods.

## Background

Flavor is a very important sensory characteristic of the overall acceptability of ruminant meat products. Volatile flavor compounds have a strong impact on the sensory properties of ruminant meat. Intramuscular fat (IMF) in meat depends on various factors and impacts meat quality and flavor. There is a higher proportion of IMF in ruminants that are fed on grains, and their meat is more tender in comparison with meat from ruminants fed on grass [1–4]. The ruminants that are finished on grains has lower concentration of α-linolenic acid as compared to the ruminants finished on grass because of the effects on the IMF of meat. The variation in the composition of fatty acids also affects meat flavor [5]. The diet of the ruminants has a direct relation with the consumers’ buying behavior as well as consumer taste [6]. Consumer acceptance behavior, taste analyses, and the buying relation are new study dimensions among all the determinants of flavor. There is also a relation between meat flavor and palatability for consumers. There are different categories that describes the meat flavor in terms of different senses like olfactory and gustatory sense, maillard reaction and lipid degradation [7]. The main objective of this review is to gather the major changes which can be the source of ruminant flavor, different factors are involved which become the main source of flavor as flavor plays a vital role in ruminants.

### Fundamentals of meat flavor

Meat flavor produced by the thermal reaction of non-volatile compounds to produce volatiles which are the major characteristics of flavor [8]. The lipids which are the sources for volatiles are responsible for specific flavor as if there is more unsaturated fatty acid changes in fatty acid deposition of ruminants and non-ruminants [9]. It is believed that the cooked meat taste is due to non-volatile compounds of fresh meat that are essential taste contributors and flavor precursors [10]. The five basic receptors in relation to taste are sweet, salty, sour, bitter, and umami. Meat flavor derives from the combinations of taste sensations as well as other sensations that are influenced by our food perception, such as color, mouthfeel, juiciness, texture, and aroma. Flavor is a multicomposite characteristic of meat tastiness. The diet of the animals influences the meat texture and flavor, because this diet alters the level of IMF and fatty-acid composition [11].

### The science of meat flavors

The Maillard reaction is important for formation of meat flavor. This reaction helps to explain carbonyl and amine reactions. Normally, meat cooked at a high temperature shows browning because when a free amino acid links with a carbonyl group forming glycosylamine, and when the latter is dehydrated and rearranged, it produces furanone derivatives, furfural, dicarbonyl compounds, and hydroxyketones [12]. These compounds add flavor to meat. As the reaction proceeds, the byproducts react with amino acids, amines, hydrogen sulfide, ammonia, and aldehydes through the processes of Strecker degradation, Schiff base pathways, and Amadori rearrangement. As the chemical reaction builds up through the process of Strecker degradation, Schiff base, or other pathways, it contributes to formation of melanoidins (high-molecular-weight brown compounds) [13].

**Fig. 1:**
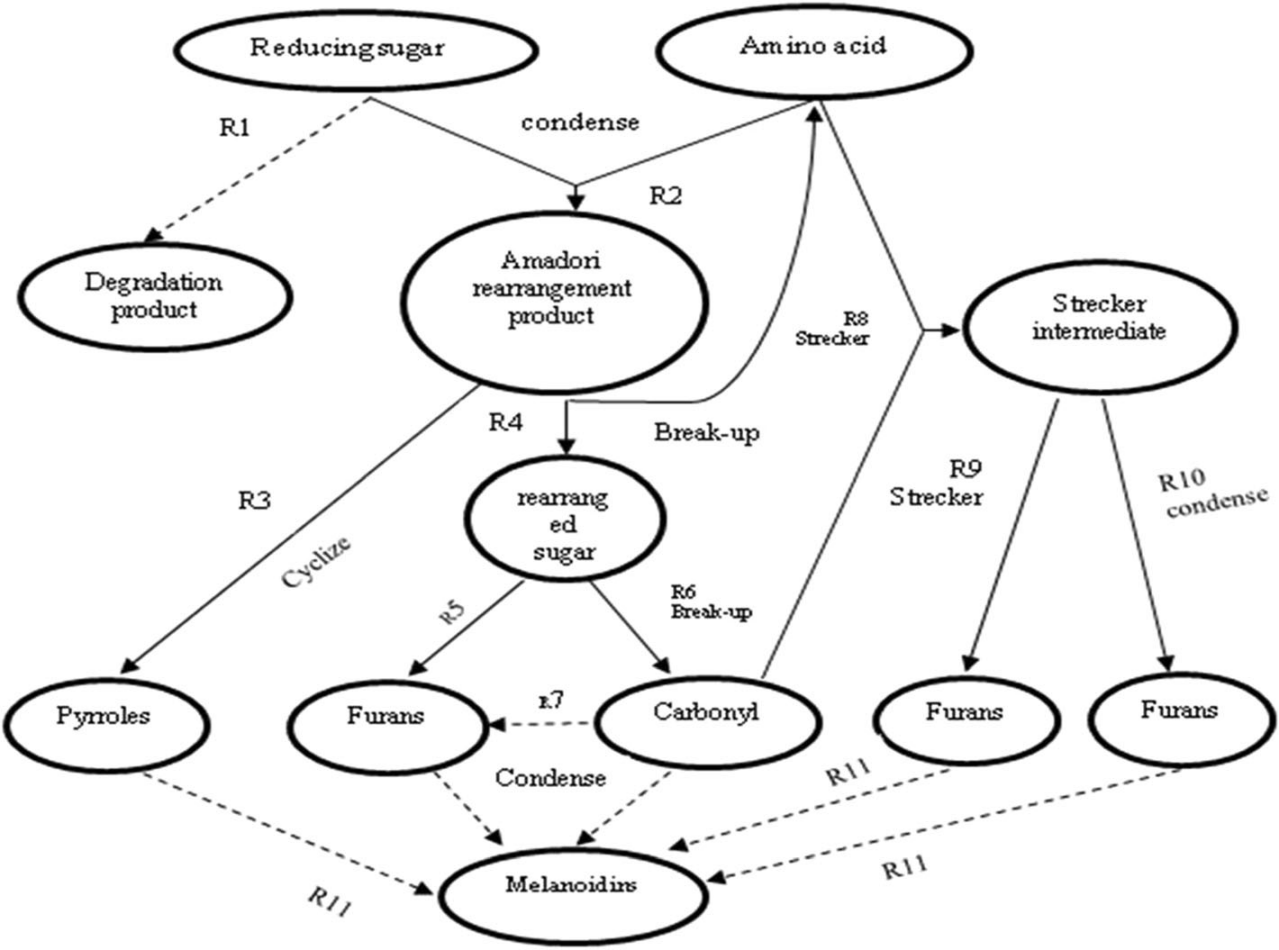
Kinetic scheme of flavor formation by Maillard reaction

In the Maillard reaction, the first step is the dehydration reaction or removal of water. A dry atmosphere is needed to support the first reaction. Strecker degradation of amino acids is linked with the Maillard reaction by a dicarbonyl compound that is produced by the Maillard reaction [14]. Aldehydes are formed by the deaminated and decarboxylated amino acids, whereas the aminoalcohol or aminoketone are converted into a dicarbonyl. Acetaldehyde, hydrogen sulfide, and ammonia are produced through the Strecker degradation because cysteine is an amino acid. The rich source of these byproducts contributes to important categories of flavor compounds, which include pyrazines (related to N), furans (related to O), oxazoles (related to N), pyrroles (related to N), thiazoles (related to S), thiophenes (related to S), plus heterocyclic compounds. Different Maillard reaction-related chemical reactions are extremely complex and lead to numerous compounds that are involved in production of flavor. The kinetic scheme of flavor formation by the Maillard reaction is shown in Fig. 1.

### Perception of the food flavor with reference to meat

The flavor of prepared foods is humanity’s greatest universal behavior, experienced by individuals of all ages in the course of daily life. Also, the flavor perception is one of complex behavior in human that involves nearly all senses especially sense of smell which engage odorous compounds generated in the olfactory pathway. It also engages the complex facial, swallowing and respiratory motor systems [15] (Shepherd, 2006).

In humans, the taste pathway ascends from nucleus of the solitary tract in the brainstem to the hypothalamus and to the taste area of the somatosensory thalamus, and from there extends to the primary taste cortex. A key fact about taste stimuli is that they elicit the most basic human emotions of pleasure (sweet) and disgust (bitter), which are not learned; they are hard-wired in the brainstem from birth Similarly, in human brain, the perceptual systems are closely linked to systems for learning, memory, emotion and language, so distributed neural mechanisms contribute to food preference [16] (Scalera et al., 1997).

The dual olfactory system of the brain involving orthonasal olfaction and retronasal olfaction. The orthonasal majorily involve brain systems involved in smell perception during sniffing in or food ingestion and retronasal olfaction uses those systems of Brain that can have smell perception during breathing out, with food in the oral cavity. Retronasal stimulation occurs during food ingestion, when volatile molecules released from the food in the mouth are pumped, by movements of the mouth, from the back of the oral cavity up through the nasopharynx to the olfactory epithelium [17] (Sun and Halpern, 2005).

### Preharvest factors affecting meat flavor

Even though a ranking of different features that affect purchase food choices has yet to be developed. There are different features involved in the purchase of meat [18]. reported that color is the major feature for the purchase of meat by the consumers. The flavor is also become reason that why various customers make meat their food of choice. The meat consumers associate the eating quality (juiciness, tenderness, and flavor) with worth, and that eating quality raises the probability of consumers’ buying meat and also increase their level of satisfaction [19, 20]. The cattle which will be not aged having meat of good color and appearance has more demand by the consumers because consumers liked the meat which has more juiciness, tenderness and flavor [21, 22]. Meat flavor is affected by a number of parameters; most of them can systematically be improved [23]. Preharvest factors that affect meat flavor comprise those that are intrinsic to the animals (i.e., sex, temperament, age, and genotype) and the factors that are related to cattle production practices (i.e., handling of animals, animal nutrition, slaughter age, live weight at slaughter, preslaughter stress, and slaughter methods). Effective preharvest management of cattle reduces inherent variation in flavor caused by cattle production methods. The impact of different preharvest factors on meat flavor is shown in Table 1.

**Table 1:** Impact on meat flavors by different pre-harvest factors

Pre-harvest factors	Impact on meat flavor	References
Animal nutrition	Various volatiles have been recognized, contributing to the discrete flavor profiles of grain-fed beef and grass fed, Animal fat plays an important role in the formation of the characteristic flavor of cooked meat.	[32, 35, 91, 92]
Feed	Feed has a vital role in affecting physicochemical as well as the organoleptic properties of meat that alternatively affects the quality characteristics	[93–95]
Sex of animal	Male has thicker subcutaneous fat and more marbling than females. That’s why male presented better quality (flavor) of meat than females	[96]
Breed of animal	Breed affects fifty-four flavoring compounds of which 75% were Maillard’s reaction products, The IMF content in different breeds of cattle varied from 0.99 to 2.72%	[10, 97, 98]
Genetic makeup	The minor flavor difference among breeds correlated moderately with the marbling factor and is considered to be heritable trait as well	[38, 99]
Age of animal	Age affects the solubility of intramuscular collagen and hence increases flavor intensity. Aged animals have higher straight chain fatty acids. Age of animal also affects the other sensory attributes.	[100–102]

### Handling of animals

Meat flavor characteristics are affected by various treatments to which the animals are subjected: handling creates stress straightaway before slaughtering [24]. Stressful environmental events, emotional stress, or whether glycogen is depleted in muscles result in unusually high pH with a dark purplish-red color of meat (the beef generally named “dark cutting”). Top sirloin and strip loin steaks because of dark cutting beef have less desirable flavor than that of the steaks cut from normal carcasses. Moreover, steaks of dark cutting beef contain more off-flavors such as “sour,” “bitter,” and “peanutty,” when compared with normal ones [25]. Off-flavor reduction and desired flavor enhancement can be achieved through implementation of managing practices that decrease preslaughter stress factors. Voisinet et al. [26], reported that temperamental cattle are more likely to yield dark cutting meat. Busby et al. [27], also found that dark cutting yields lower marbling scores in carcasses, and both of these negatively affect the flavor of meat. Heifers are more impulsive than steers and thus produce carcasses with the characteristic dark cutting [26]. Heifers are much more temperamental than steers according to the cattle temperamental score, which significantly correlates with various characteristics of longissimus muscle together with sensory panel and muscle color ratings for flavor and tenderness. The cattle with excitable temperament possesses much darker muscle color, greater muscle pH, and lower sensory ratings for tenderness and flavor in contrast to the cattle with less excitable temperament [28]. These observations highlight the significance of placid management of slaughtered cattle throughout the transport and right before harvest for assessment of final produce quality. Quality grades of carcasses were compared in terms of temperament, and the results revealed that cattle classification named “docile” yields a greater proportion of carcasses with the rating “U.S. Choice or Prime” (74%) as compared to the cattle with “aggressive” temperament (58%). The incidence of Certified Angus Beef® rating for “docile” cattle is 29%, which is roughly twice the rate of 14% for cattle with “aggressive” temperament [27]. Some studies revealed that the cattle temperament is moderately heritable [29]. Therefore, effective sorting of cattle by docility could have advantageous effects on numerous quality characters of meat: tenderness, color, flavor, and marbling.

### Ruminant nutrition

Diet is the chief feature affecting ruminant meat flavor. Different tissue elements are influenced by diet and affect the flavor, with fatty acids being a significant factor [30, 31]. Meat of forage-fed ruminants contained more linolenic and additional n-3 polyunsaturated fatty acids, while meat from grain-fed ruminants contains more oleic, linoleic acid, and other n-6 polyunsaturated fatty acids [9, 32]. A comparison of the effects of forage versus grain feeding on the fatty-acid composition and flavor of meat revealed that a noteworthy percentage of the dissimilarity in the score of flavor between forage-fed and grain-fed ruminants is due to the greater concentration of oleic acid in grain-fed ruminant meat in contrast to greater levels of linolenic acid in the forage-fed ruminant meat. Sensory panelists label the less desirable flavor of the ruminant fed with forage as “fishy,” “grassy,” “gamey,” or “milky,” while the “ruminant fat” flavor usually associated with grain-fed ruminant [30, 31]. Elevated levels of ruminant linolenic acid have been reported to create “fishy” or “grassy” flavor [33]. The compound with a “grassy” flavor was found to be phyt-2-ene. In contrast, δ-hexadecalactone, 2-lactones, and δ-tetra-decalactone are inversely associated with the “grassy” flavor in ruminant [34]. Lactones are linked to the “roasted flavor” of meat of grain-fed ruminant, while triterpenoids are described as “gamey/stale” and are associated with an off-flavor by sensory panelists of grass-fed ruminant. Several volatiles were isolated that correlate with flavor and differ between grain-fed and grass-fed beef (and can efficiently imitate the specific “ruminant fat” flavor of ground grain-fed beef via addition to the diet of forage-fed cattle) along with the low levels of toluene, m-xylene, and pentanal [31]. Various volatiles have been identified that contribute to the discrete flavor profiles of grain-fed and grass-fed ruminants [9, 32, 35].

### Genetic makeup (breed)

The breed of animals affects flavor, eating quality, and fat percentage: the animals with high fat content have superior scores [36, 37]. This observation indicates a positive effect of fatness on the eating quality. The nongenetic effects, including diet before slaughtering, greatly affect meat flavor and can have nonadditive as well as additive effects with genetic factors [38]. Genetically derived flavor intensity of beef [39, 40] usually shows weak effects, suggesting that 10% or less of the difference in flavor may be attributed to additive genetic outcomes. Wheeler et al. [41], reported that meat flavor may be reasonably genetic, pointing to possible selection (sorting) for enhanced flavor, which is unfeasible due to the complicated procedure and costs of phenotype assessment. In addition, a broad-spectrum comparison of cattle breeds has revealed some important differences in the flavor of meat [41, 42]. The minor flavor differences among breeds correlate moderately with the marbling factor and are considered a heritable trait as well [38, 43]. Additionally, different rates of genetic correlation with marbling, % IMF, and flavor have also been documented for beef [38, 41]. Ultimately, the selection of cattle on the basis of marbling probably would result in the improved resultant flavor. The effect of different species and breeds on meat flavor is shown in Table 2.

**Table 2:** Effect of meat flavor by different species and breeds

Breeds	Impact on meat flavor	References
Lambs	The research showed that if lamb consumed forage 4 to 6 weeks prior to slaughter may affect the flavor of meat and acceptability of consumers	[103]
Beef	The beef which was forage finished having less beef flavor and more off-flavor as compared to concentrate finished beef. There was 36% reduction in lipids in beef fed with grass than conventional beef	[104, 105]
Cattle	There was heterogeneity found in cattle in different countries due to pasture types and their breeds that affects the nutritional composition in meat and also affect the sensory attributes specifically the flavor.	[106]
Goat	Goat meat contained high amount of PUFA and less saturated fatty acid and less amount of aldehydes detected and good flavor of meat	[107]
Goat	Fatty acids play a vital role influencing the goat meat flavor. 4-ethylocatanoic was the specific fatty acid that produced strong flavor particularly in goat.	[108]
Sheep	The feeding system plays a vital role in the final cooked product in terms of flavor. For example if sheep consumes brassica then it will effect on the flavor of meat and its products.	[109]
Steers	The steers fed on forages instead of concentrates having more contents of n-3 fatty acids and conjugate linoleic acids and having more flavor.	[104, 110, 111]
Heifers	Heifers with different breeds like Aberdeen Angus × Friesian and Belgian-Blue × Friesian have better flavoring profiles as compared to bulls	[112, 113]
Bulls	Among bulls breed, Holstein has better flavoring profile as compare to others like Limousin. Among heifers, bulls and steers, bulls have worst sensory profiles including flavors.	[113]
Steers	Steers had the best sensory qualities including the flavor. The steers has higher flavoring profiles as compared to heifers. The breed Charolais×Friesian of steers has the best flavor profile than breed Belgian-Blue×Holstein.	[113]
Beef	Juiciness has positive relation with IMF as well as marbling. There was negative relationship with the magnesium and beef flavor but some other minerals have positive relationship with flavor and juiciness.	[114, 115]

### Weight and age at slaughter

Older-animal meat is darker, intense in flavor, and firm, while the meat from young animals shows relatively increased levels of tenderness, lower flavor attributes, and lighter color. In one study, the measurements of color, texture, pH were found to be affected by carcass weight: the heavier lambs had less tough meat, with higher pH and darker color [44]. A study revealed the sensory attributes of three breeds at different ages and weights (10 to 12, 20 to 22, and 30 to 32 kg). Meat flavor and odor severity increased with tenderness, juiciness, and weight depending on the breed of the animal. In fact, tender and juicy meat was obtained from Spanish Merino (heavier animals) and Churra breed (dairy, lightest lambs). When three samples of meat from the same breeds were examined, the lambs 10 to 12 kg yielded the most tender meat with better flavor in comparison with heavier lambs (tough meat). The slaughter weight of a light carcass (7.6 kg) and that of a heavy carcass (11.4 kg) in milk kids were analyzed, showing important effects on the meat quality. Light kids yielded higher firmness on the texture basis, but light-kid meat was juicier and more tender than that from heavy ones, with higher species odor and fibrosis [45].

### Animal sex

Meat from whole males may be different in flavor characteristics and different in tenderness and may be tougher than that of castrated females or males. Female animal meat varies in fat and connective-tissue proportion, depending on the association of puberty onset and growth. The level of breakdown products of testosterone, higher levels of androstenone and skatole that form in the hind gut cause boar taint. The gender effect on flavor and odor is less clear; some studies showed no variation in beef flavor intensity and quality between steers and bulls [46, 47]. Nonetheless, some studies reported the flavor with lower intensity and chiefly more abnormal flavor in the meat of bulls [48, 49]. In another study, the beef flavor in bulls was found to be stronger than that present in heifers, with greater livery flavor and odor. There are small variations between heifers and steers in some studies. Conclusively, the intensity of beef flavor may be enhanced, and unusual flavor may be amplified in bulls in contrast to heifers and steers [50].

### Preslaughter stress (PSS)

PSS has adversative effect on the quality of meat that leads to dry, firm, and darker (DFD) meat in cattle. PSS occurred due to a number of exogenous and endogenous factors that affects the post-mortem biochemical changes that may cause the conversion of muscle into meat. The PSS in cattle may cause the formation of DFD meat due to lessening the muscle glycogen reserves and lactic acid accumulation that alters the normal process for acidification of meat during post-mortem and affects the meat quality like tenderness, juiciness and flavor [51, 52]. The DFD meats becomes when post-mortem pH determined after 12–48 h will be higher than 6.0 [53] which are more common in bulls as compared to heifers or steers [54]. In addition, pH has an inverse relation with flavor of cooked beef [55] due to the concentrations of sugar phosphates having low amount in DFD meat, ultimately yield a low level of Maillard reaction products. In another study, three groups were isolated, with the pH of less than 5.8, 5.8, and 6.1 and more than 6.1. The panelists showed that the percentage of “foreign” flavors increased from 5 in the group of low pH to 11 in the group with high pH. In another study, Millard reaction products were found to have an inverse relation with water content of beef.

### Postharvest factors influencing meat flavors

The most important factors affecting meat preference is the flavor of meat. Flavor involves two sensations: taste and aroma. The perception of flavor is often more about texture than flavor molecules. Texture changes as a result of protein coagulation, fat melting, collagen turning into gelatin, when moisture is driven off from the surface, and when starches turn stiff and crunchy [56]. These affect the “mouth feel” as well as flavor. The flavor caused by different cooking methods in meat is very important for producers and consumers. Formation of off-flavors due to lipid oxidation lowers the meat quality [57]. Flavor is also affected by several postharvest factors, like pH, temperature, protein, fats, glycogen, fatty acids, marbling, and by different cooking methods. The effects of different postharvest factors on meat flavors are presented in Table 3.

**Table 3:** Impact on meat flavors by different post-harvest factors

Post-harvest factors	Impact on meat flavor	References
Fats and Fatty acid composition	The aroma of meat affected by crude fat contents. The fatty acid contents of meat varies greatly depending on an animal’s diet and higher amount of PUFA is beneficial for the CVD patients.	[116–119]
Proteins	The proteins in meat can be hydrolyzed by natural proteolytic enzymes during storage and also during aging due to which there is production of peptides and free Amino acids. The role of small molecular weight peptides is not well defined but it is accepted that it may helpful in met flavor development	[120–122]
Marbling	The term, marbling, originates from the beef industry and is considered to be highly desirable to achieve tenderness and desirable flavor and juiciness.	[122–124]
Aging	Postmortem aging enhances the tenderness by the enzymes as well as positive impact on the flavor development.	[125, 126]
Temperature	Pre-rigor excised muscle, +/− wrapped (or restrained) holding for 24 h at 15 °C or for 7 h at 37 °C having more tender meat with pale color, sweaty odor and some bad flavor observed	[127]
pH	pH plays an important role in Maillard reaction regarding the impact of meat flavor. As the pH increases, polymeric nitrogen-containing compounds like pyrazines compounds also increases which affect the flavor	[9, 128]
Irradiation	The flavor and aroma of meat also affected by production of free radicals during irradiation	[129, 130]

### Fats and fatty-acid composition

Fat is the major contributor to the flavor development in meat. There is variation among species in flavor development. Different flavors of breeds result from the fatty components. Fatty tissues give the meat specific flavor attributes. Fat is one of originators of flavor due to different kinds of fatty acids. As the fat melts, it produces flavors [58]. It is generally known that the composition and the amount of polyunsaturated fatty acids in ruminants mainly depend on diet. From polyunsaturated fatty acids and oxygen, peroxides are synthesized via the free radical chain mechanism. Lactones, aldehydes, hydrocarbons, and ketones are formed by oxidation of meat and create unwanted, rancid off-flavors. Antioxidant compounds control the oxidation in muscle tissue. Grain-fed beef is more susceptible to lipid oxidation than grass-fed beef, and this effect is due to the increased levels of vitamins A, C, and E, flavonoids, and carotenoids present in forages.

Polyphenols or vitamin E supplements that are consumed by grain-fed animals act as antioxidants during the finishing period. Lipids play an important role in flavor development. Shahidi [59] reported that during production, handling, and thermal processing, lipids act as a solvent on volatile compounds in meat. Beef flavor is influenced by certain compounds produced during thermal oxidative changes and rejoin lean tissue to produce distinctive flavoring compounds. Mottram and Edwards [60], reported the relations among 14:1, 16:1, 18:0, 18:1, 18:2, 18:3 fatty acids and desired beef flavor. On the other hand, species flavor depends mainly on ketones, saturated aldehydes, fatty acids, and unsaturated aldehydes, which all play a major role in meat flavor [31]. The meat flavor of different species is expected to have similar mechanism having sugars and amino acids in their meat after heating but flavor can be different. The meaty flavor is generated by the precursor supplied by lean tissues common to all cooked meats. Upon lipid degradation, aldehydes have typical features in certain species. For example, 2-alkenals such as hexenal, heptenal, octenal, and nonenal as well as aldehydes, including octanal, nonanal, and decanal are linked with both a particular flavor and aroma. Because there are differences in their digestive systems, fatty-acid deposition between ruminants and nonruminants is different. There are higher levels of polyunsaturated fatty acids in the triglycerides of beef or lamb [9]. Therefore, the lipids are influenced due to the differences in the resulting carbonyls and fatty-acid profile [61]. Primarily, oleic and linoleic acids of unsaturated fatty acids are present in triglycerides of red meat and poultry. However, phospholipids contain relatively higher levels of linolenic and arachidonic acid. Linoleic and arachidonic fatty acids auto-oxidize and form 2-nonenal, 2,4-decadienal, 1-octen-3-one, 2,4-nonadienal, and 2-octenal through 9-hydroperoxide and 11-hydroperoxide, respectively. A meaty flavor is contributed by 2-nonenal and 2,4-decadienal [62]. As a result of oxidation of arachidonic acid, the most intense aroma compound is trans-4,5-epoxy-(E)-2-decenal, followed by 1-octen-3-one, 2,4-decadienal, 2,4,7-tridecatrienal, and hexanal.

Palatability, including flavor, is affected by fat content. Most United States consumers prefer increased IMF as well as fat flavor. For the United States consumer, the lowest acceptable level of IMF is approximately 3% in beef [63]. There is a negative effect on flavor acceptability and perception if the fat level is above 7.3% in meat. Marbling score and grading are less affected by flavor desirability of the top round steak [63]. The influence on tenderness and juiciness is greatly affected by IMF depending on the studied species of sheep; a sensory panel valued the meat with more IMF or marbling much more highly. As the IMF level in meat increases, the shear force value decreases, suggesting that the IMF level is not directly related to the tenderness point. In relation to cancer and heart disease, the fatty-acid composition of meat is very important, with implications for human health. Similarly, the features of meat like juiciness, flavor, firmness of the fat, and shelf life are affected by the fatty-acid composition [33]. Similarly, the characteristic of polyunsaturated fats termed conjugated linoleic acids (CLAs) is also important. The abbreviation CLA is a collective term used for all geometric and positional isomers of linoleic acid with conjugated double bonds. Among them, there are two major isomers: a) CLA trans-10, cis- 12, and b) CLA cis-9, trans-11 (rumenic acid), occurring in dairy products and ruminant meat (in amounts of approximately 10–25% and 75–90% of total CLA, respectively). Antiadipogenic, antidiabetic, and anticarcinogenic effects, and positive effects on the immune system are major benefits of CLAs.

Via the partial biohydrogenation of conjugated fatty acids, CLAs are formed in the rumen, because in the meat of ruminant animals, they are found at high concentrations, e.g., in sheep. The chief features affecting biohydrogenation are the concentrate ratio, the forage, ruminal pH, and the kind and level of fatty-acid intake. For instance, linoleic and linolenic acids significantly contribute to low ruminal pH. Increasing CLA cis-9, trans-11 content in meat and meat products is a strategy to enhance C18:1 trans-11 uptake in the duodenum. Miller [64] reported that this is because CLA cis-9, trans-11 is also produced by endogenous conversion of C18:1 trans-11 (trans-vaccenic acid) by the enzyme Δ-9-desaturase in adipose tissue and the mammary gland, suggesting that CLA cis-9, trans-11 synthesis increases linearly with an increase in the C18:1 trans-11 content of the diets of human subjects. The rate of C18:1 trans-11 conversion to CLA cis-9, trans-11 ranges from 19 to 30% in humans to 5–12% in rodents.

### Marbling and types of muscles and muscle fibers

The marbling and the types of muscles and muscle fibers affect the meat quality and alternatively affect the flavor of meat. There are four types of muscle fiber present in the skeletal muscle of adult animals, which include fast oxido-glycolytic or type II A, slow-oxidative or type I, fast glycolytic IIB, and IIX. Fibers of these types are present commonly in meat muscles, and their proportions can determine most of the muscle’s metabolic properties in different muscles. Therefore, postmortem metabolism of muscle is an important parameter to analyze; quality of fresh meat may or may not be affected by cross-sectional area of muscle fibers, and depends on the total fiber number and proportions of fiber types. The characteristics of muscle fiber are affected by different factors including hormones, muscle location, breed, gender, growth performance selection, and diet [65, 66].

In addition, in many countries, the meat industry showed interest in promotion of different types of muscles. Nonetheless, the quality characteristics and factors of individual muscles are mostly unknown. Skeletal muscles contain different types of fiber, which are influenced by various factors including muscle type, breed, hormones, and age. Meat quality is affected by characteristics of muscle fiber including marbling, water-holding capacity, texture of meat, and color. Generally, marbling strongly affects meat quality. Mainly, marbling is a significant feature in the meat industry, whereby consumers judge the quality of meat, and may also affect the flavor. The effects of muscle fiber characteristics on meat quality are studied broadly in ruminants, although there are some reports regarding poultry and swine [67].

Muscle fiber (myoglobin, Mb) characteristics affect marbling and flavor in meat. The rate of Mb oxidation and Mb content are unique for each muscle type; manufactures maintain the fraction of red muscle fibers high, which results in an increase in Mb content and redness of meat. As the ratio of Type I fiber increases, the stability of color decreases, resulting in shifting of the meet muscle fiber color to brownish. In young bull muscle, the fast-twitch glycolytic (IIB) fiber content correlates with lower water-holding capacity and higher lightness. Hypertrophy of fast-twitch oxido-glycolytic fibers (IIA) is significantly disadvantageous for the water-holding capacity. Size of the fiber bundle and muscle growth potential are affected by the size of muscle fibers; big size results in noticeable coarseness of cross-sections of meat [66].

Connective tissues and their proportion together with IMF also affect the characteristics of muscle fiber [68]. In porcine longissimus muscles, a well-built positive genetic correlation is observed between IMF and the cross-sectional area of fibers (CSAF) proportion; it is also well documented that in porcine longissimus muscle, the size and proportion of the fibers of type IIB positively correlate with IMF. IMF correlates negatively in beef muscle with white Mb but is related positively to the percentage of red Mb. Normally, it is reported that more IMF is present in red oxidative muscles as compared to white glycolytic muscles; but one study showed that there is no correlation between fiber type composition (FTC) and IMF and recommended that both of the characteristics can be manipulated [66].

FTC of muscle is linked to glycolysis, the pH decline rate, and protein metabolism (proteolytic degradation). Highly glycolytic fibers are fast-twitch IIB fibers, and their metabolism results in high-speed metabolism in the early protein-metabolic period. Rapid glycolysis is enhanced in muscles if they contain fast-twitch glycolytic fibers as a major component, and this situation results in a rapid decline of pH in muscles. Therefore, the proportion of type IIB fiber positively correlates with the R-value and negatively correlates with muscle pH (adenine/inosine ratio); this phenomenon allows for evaluation of ATP reduction during the early postmortem period. Nevertheless, an increase in the ratio of fibers of type I in muscle reduces the extent and rate of decline of postmortem pH. FTCs are the cause of variation in postmortem muscle properties, hence affecting meat tenderness. In addition, fast fibers of type II are more vulnerable as compared to type I slow fibers in the case of early postmortem proteolytic degradation. Some researchers stated, however, that tenderness increases with the increasing ratio of fiber of type I, while it decreases with the increasing ratio of type IIB fibers in the muscles of cattle [69, 70].

The effects of muscle fiber characteristics on postmortem aging have also been suggested as a chief determinant of meat quality. Fast-twitch glycolytic fiber positively correlates with postmortem aging in cattle and with tenderness. In slow-twitch oxidative muscles, the rate of aging is slower as compared to fast-twitch muscles. The ratio calpain/calpastatin is lower in slow-switch oxidative muscles than in fast-twitch glycolytic muscles. To some extent, these phenomena could explain the faster rate of aging in glycolytic muscles. Fast-twitch fibers are believed to have a more widely developed transverse tubule system, sarcoplasmic reticulum, and slim Z-band in comparison with slow-twitch fibers. The protein responsible for the Z-band in fast twitching fibers is more prone to early postmortem protein degradation as compared to slow twitch fibers. IMF content strongly affects the juiciness and flavor of meat, which is positively related to the proportion of type I fibers in muscles. Phospholipids are the chief determinant of cooked-meat flavor, and the level of type I fiber is linked with it. Meat juiciness also positively correlates with type I fibers, even though one study has clarified the notion that more IMF is present in red oxidative muscles as compared to white glycolytic muscles [9].

### Impact of different cooking methods and seasonings on meat flavor and their health effects

There are many cooking methods that have an impact on meat flavor. There are many volatile compounds produced during cooking.The high-heat treatment involves production of volatile flavoring compounds due to the Maillard reaction [71]. The effect of different cooking methods on meat flavors is shown in Table 4.

**Table 4:** Impact on meat flavors by different cooking methods

Cooking methods	Impact on meat flavor	References
Pressure cooking/Microwave cooking	Microwave treatment, despite using shorter time and lower temperature also promotes lipid oxidation. The desirable quality attributes were developed better with pressure cooking than microwave cooking technique	[131, 132]
Roasting	When different cooking methods were compared, roasting, which uses high temperatures for a long time, produces an increased lipid oxidation compared to other methods	[133]
Frying	Frying is one of the oldest methods of food preparation and improves the sensory quality of food by formation of aroma compounds, attractive color, crust and texture, but oils or fats can change the fatty acid composition of meat and suffer oxidation	[134, 135]
Curing	Improves the meat flavor and also enhances the stability of meat and meat products	[136, 137]
Smoking	Smoking treatment is helpful to develop flavor. That flavor comes from the wood and high use of smoked meat can be carcinogenic	[73, 74]

Warm-off flavor means undesirable flavors that result from flavor changes and deterioration in reheated, precooked, or chilled-stored meat. There are different kinds of tastes and odors of warm-off flavors such as rancid, bitter, stale, cardboardlike, painty, and liverlike off-flavor. These flavors are the main factors that affect the sensory and eating quality of meat. Oxidation of membrane phospholipids is the major cause of warm-off flavor found in cooked meat. Byrne et al. [69], reported that the process of lipid oxidation is associated with the warm-off flavor. The meaty flavor is also reduced by the development of warm-off flavor due to lipid oxidation [70].

There are many dry-heat cooking methods that affect the flavor of meat. In broiling and grilling, the cooking time is very crucial during preparation of products like streaks; kababs are cooked at higher temperatures. Studies show that during roasting, many of the flavoring compounds are lost as compared to boiling, which preserves many flavoring compounds such as heterocyclic compounds, e.g., pyrazines, thiazoles, and oxazoles [71]. Frying is mostly used for the uniform meat cuts, but there is a risk of flavor loss depending upon the conditions of frying. Velveting is a cooking technique that enhances the texture of fried meat cuts by means of cornstarch marinade [72].

Moist-heat cooking methods are also helpful for flavor preservation. In these methods, a liquid is used in varied quantities to preserve flavor at various high temperatures and with varying duration. Braising is slow and gentle cooking in a liquid. The moist-heat methods involve low heat in a tightly covered pan, to which liquid has been added. The moist heat methods solubilize the collagen and produce natural meat flavors in less tender cuts, and the steam produced by the liquid converts the tough collagen into tender gelatin. Sometimes, the meat flavor compounds leach into the cooking liquid creating delicately flavored meat during long, slow cooking in moist heat. The quantity of water makes a big difference between cooking by braising/pot roasting and liquid/stewing [71]. Smoking is also an important method that is used for cooking, flavoring, and preserving the food by exposing it to smoke mostly from wood. *During prolonged exposure to smoke, the meat surface will acquire a smoky flavor.* The strength of the flavor depends upon the time and density of the smoke [73, 74].

The high-pressure treatment is very useful for preserving the sensory quality, especially flavor and taste of meat products [75]. reported that if meat is treated with pressure of 300 MPa, then it has better flavor and taste in comparison with treatment with 450 MPa. It is believed that when beef are subjected to treatment at 400 MPa, then the level of some alcohols and aldehydes significantly decreases and the production of 2-butanone and 2,3-butanedione increases. Due to the increase in the level of volatile compounds, this approach has a good impact on flavor, particularly the aroma [76].

Meat cooking plays a crucial role in affecting the health of an individual. As discussed earlier, different cooking methods have different types of health effects. The temperature and the time of the cooking (thermal conditions) are the important parameters. Lower temperature of cooking is beneficial as it require less energy consumption but for the safety of meat, it require final internal temperature of 65–80 °C [77]. Roasting required high temperature for a long time and formation of lipid oxidation products also higher when compared to other methods. On the other hand, microwave treatment require less time but also produced lipid oxidation [78, 79]. Heterocyclic aromatic amines (HAA) also produced in meat when cooked at higher temperature and after cooking these compounds remained in the final product [80].

There are many factors which influenced the formation of HAAs in meat during cooking including, cooking methods, cooking time, animal flesh type, and contents of amino acids as well as fat. When meat cooked at higher temperature for long time, the concentration of HAAs were found to be higher [81]. There are many health effects caused due to HAAs during cooking methods. Different types of cancers caused by mutation of genes and abnormal growth of cells are due to the formation of HAAs in meat. These carcinogens are produced due to higher temperatures in meat cooking methods [82–84].

Seasonings are the plant parts used as food flavoring and the development of processed spices has resulted an important support industry for food processing enterprises to meet consumer demands. The seasoning mixtures are formulated to serve as flavoring agents for processed meat products and the major groups of seasonings include natural spices, herbs and vegetable bulbs. Natural spices includes dried rootstocks, barks, flowers or their parts and fruits or seeds of different plants. The most important natural spices used in processed meat products are pepper, paprika, nutmeg, mace, cloves, ginger, cinnamon, cardamom, chilli, coriander, cumin and pimento [85]. Seasoning are mainly used as flavoring and coloring agents in meat and meat products however, their addition can also increase the safety as well as preservation of the products over a long period of time [86].

Various studies reported that role of different seasonings to improve the flavor profile of the meat along with preservation and quality retention of the product. A study by [87] documented that addition of garlic compounds (diallyl sulfide, diallyl disulfide, s-ethyl cysteine, and n-ethyl cysteine) in ground beef improved color and microbial safety as well as decreased the rate of oxidation without compromising the sensory attributes of the products. Similarly, cinnamon, clove, fennel, star anise, and pepper were studied as possible natural antioxidants by Dwivedi et al. [88]. They narrated clove (0.1%) and the other spices (0.5%) in cooked ground beef had effective antioxidant effects compared to control without seasonings. Also, the addition of above mentioned seasonings also reduced the generation pf rancid odor and flavor, and imparted different spicy notes to the samples, e.g. licorice and spicy flavor from adding fennel or star anise and peppery and hot from adding pepper. The study also reported positive influence of seasonings on beef products.Likewise, some seasoning are used to extract the major compound present in them such as sage or oregano, when their extracts are added to beef patties can induce bioactivity after cooking and digestion, thus can be used as functional ingredients [89]. The above mentioned studies reported the role of various seasonings that can influence the flavor of the meat products along with preservation and shelf life extension of the tested products.

### Personal predisposition and meat flavor

Few studies reported the role of seasonings addition on beef flavor that can affect the consumer choices for the meat products. The personal predisposition of the food especially meat and meat based products varied around the globe as US consumers like beef products with higher initial flavor impact, brown/roasted, and salty characteristics than other countries peoples. In this regard, a study was to determine the most popular beef seasonings used in Argentina, United States (US), and Spain. After establishing the typical cooking methods and seasonings, descriptive analysis was used to determine the differences in the main flavor attributes, particularly the impact on beef characteristics. Findings indicated average US consumers would prefer beef products with more initial flavor impact, brown/ roasted and salty characteristics than Argentinian or Spanish consumers. They also reported that seasonings addition influenced major attributes but the major attributes were affected by cooking method. This study also indicated the personal predisposition of the consumers regarding the likelihood as well as acceptability for the meat products [90].

## Conclusions

Meat flavor is a combination of taste and aroma and is one of the major parameters that affects acceptance of meat by the consumer. Several factors affect the meat flavor. The Maillard reaction plays a vital role in the development of meat flavor mediated by volatile and nonvolatile precursors. The conditions and methods of meat cooking are crucial for flavor development because due to thermal reactions, many volatiles are produced that contribute to flavor. It has been demonstrated that high temperature produces a better aroma and flavor due to Maillard products. The high temperatures induce synthesis of compounds that favor mechanisms involved in the inflammatory response and oxidative stress. These processes involved in the development of disease. The pre- and postharvest factors are highly influential for meat flavor, but for many of these factors, clarification is needed regarding their effects on meat flavor. Thus, further research is needed on the chemistry and formation of compounds via Maillard reaction products that affect meat flavor.
